# Supplementary material for: BAllC and BAllCools: efficient formatting and operating for single-cell DNA methylation data
Source: Bioinformatics. 2024 Jun 21;40(7):btae404. doi: 10.1093/bioinformatics/btae404 (PMC11216754; doi:10.1093/bioinformatics/btae404)
Supplement: btae404_Supplementary_Data [file btae404_supplementary_data.zip › SI.legends.docx]

**Supplementary Information of “BAllC and BAllCools: Efficient Formatting and Operating for Single-Cell DNA Methylation Data”**

Table S1. Specification for the cmeta format

Figure S1. The time needed to merge single cell DNA methylation data is remarkably reduced with the BAllC format and the BAllCools

Figure S2. The BAllC format and BAllCools provides flexible data query. **A**. DNA methylation data of genome regions of interest can be easily queried. **B**. and **C**. Further filtering based on context of cytosines is enabled
