# Supplementary material for: BAllC and BAllCools: efficient formatting and operating for single-cell DNA methylation data
Source: Bioinformatics. 2024 Jun 21;40(7):btae404. doi: 10.1093/bioinformatics/btae404 (PMC11216754; doi:10.1093/bioinformatics/btae404)
Supplement: btae404_Supplementary_Data [file btae404_supplementary_data.zip › SI.washu.tutorial.docx]

**Supplemental material**

**Tutorial: Visualizing BALLC format files on the WashU Epigenome Browser**

This tutorial shows you how to visualize the methylation data encoded in the BALLC format on the WashU Epigenome Browser.

1. Open the Browser at <https://epigenomegateway.wustl.edu/browser/>, here we chose the human hg38 as our genome and kept only the refGene track.

| 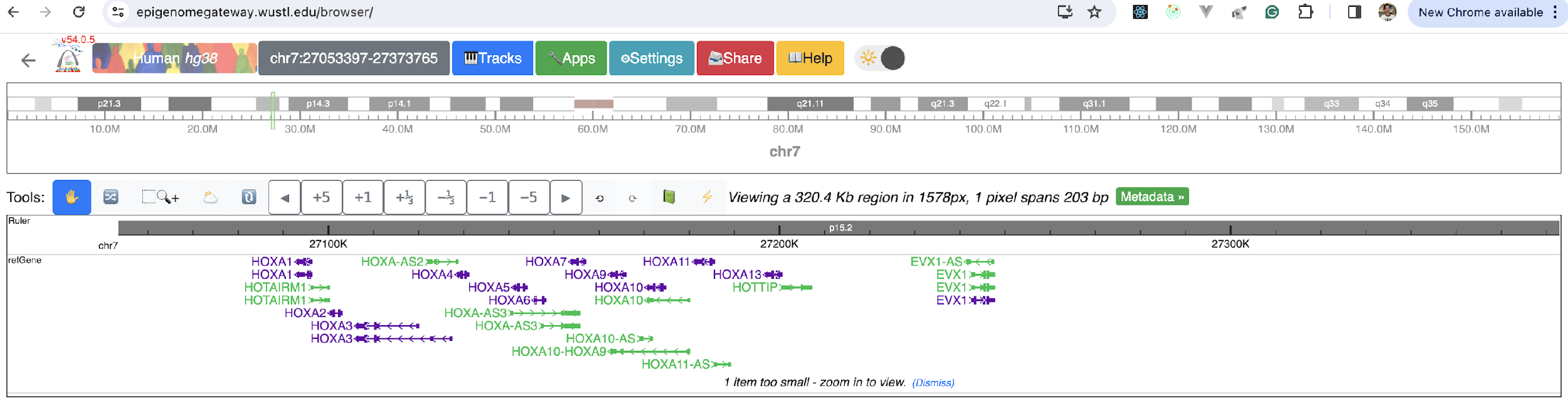 |
| --- |

1. Add a BALLC file hosted from your web server using the Remote Tracks open dialog:

| 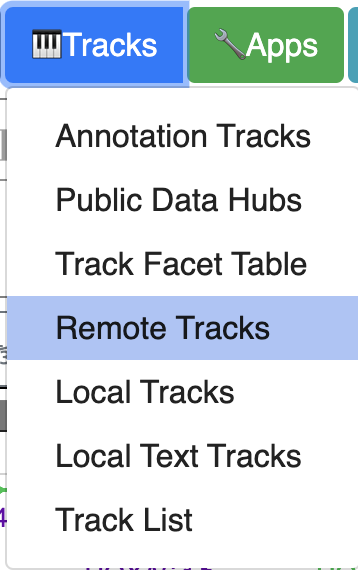 |
| --- |

| 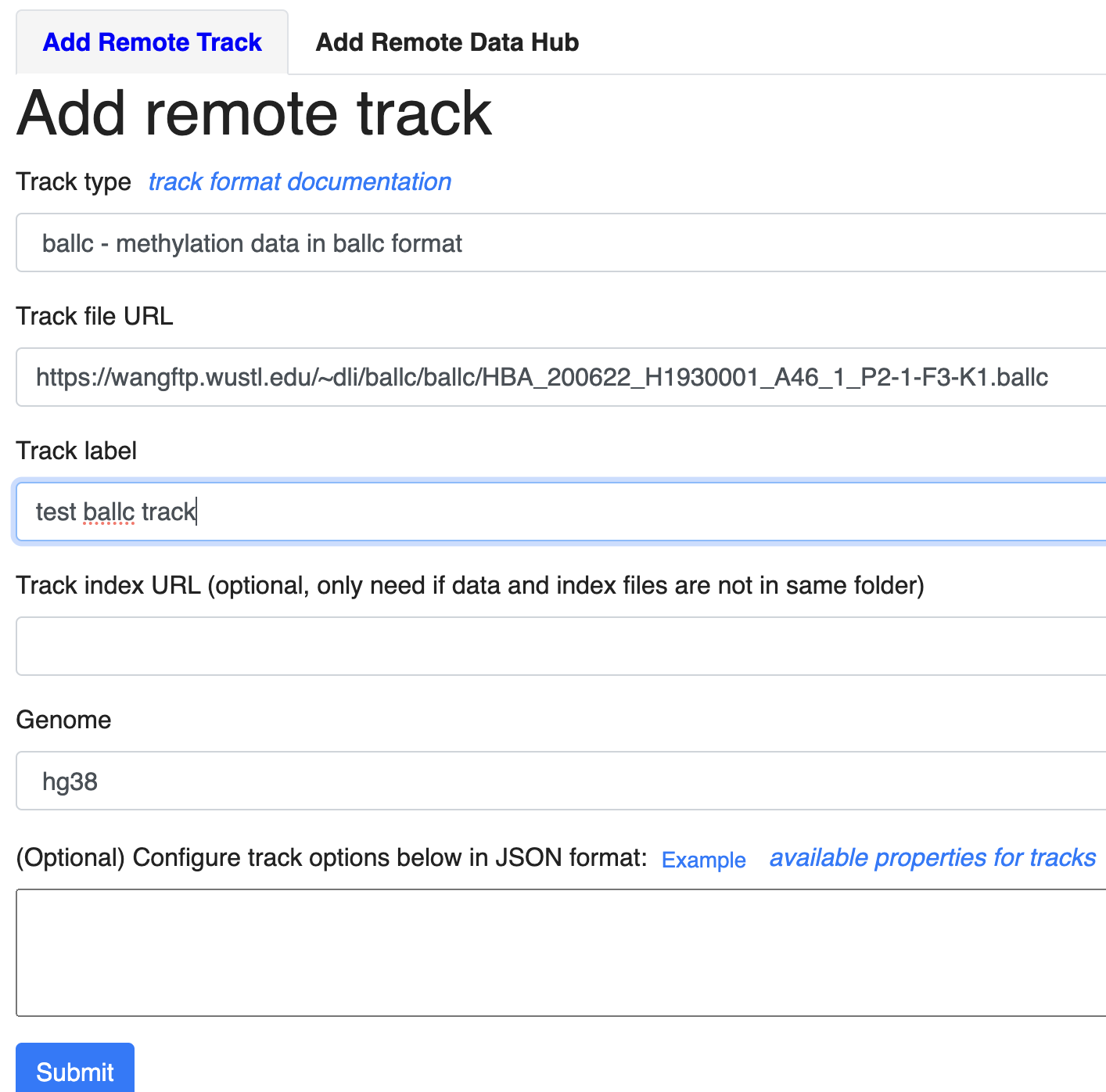 |
| --- |

1. Click the Submit button, the BALLC file will be display in the Browser:

| 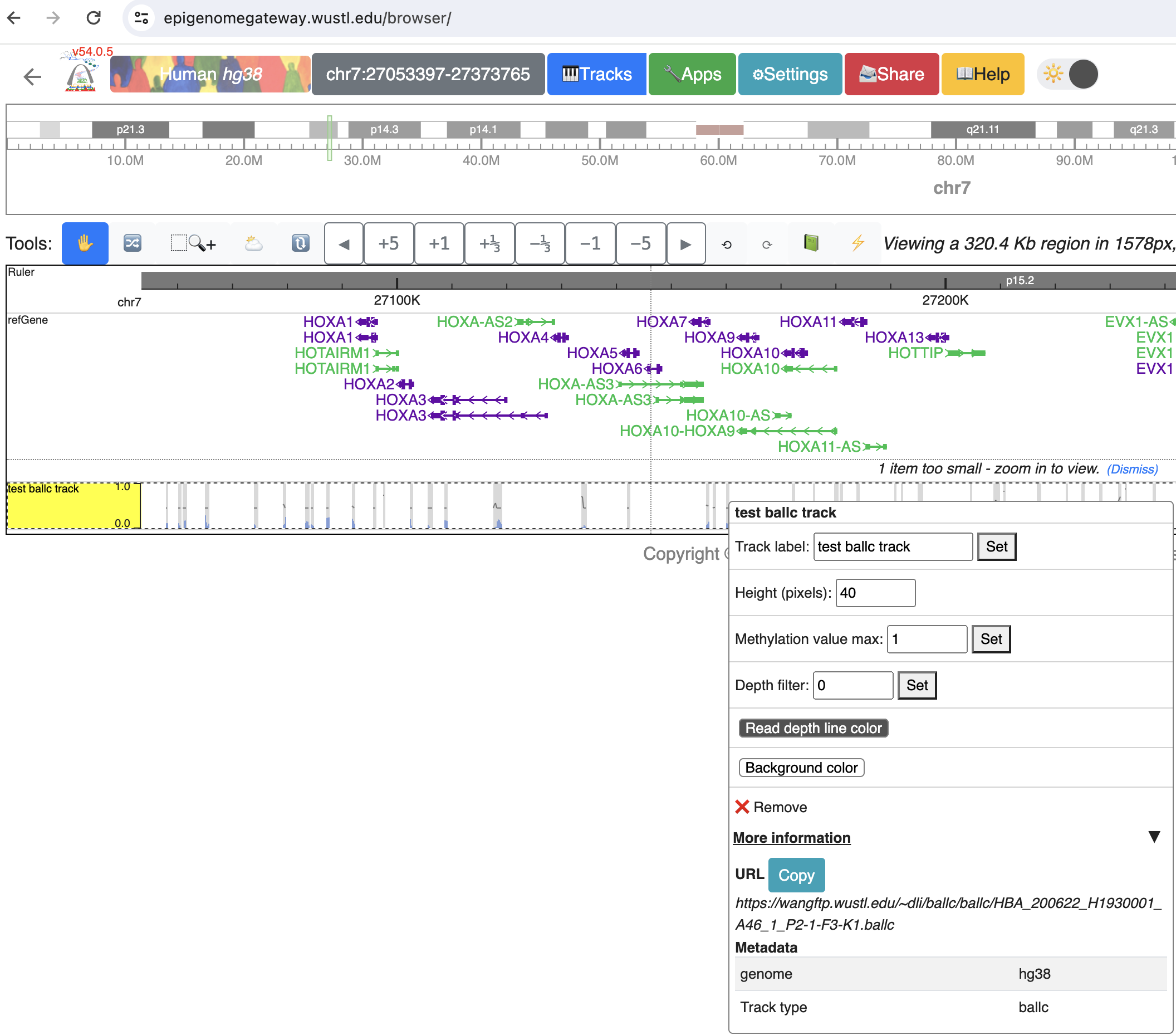 |
| --- |

Each bar in the track indicates methylation signal in the region summarized from the CpG sites in it, blue bar indicates methylation percentage from 0 to 1, black line represents read depth/coverage.

If we zoom into base pair level view:

| 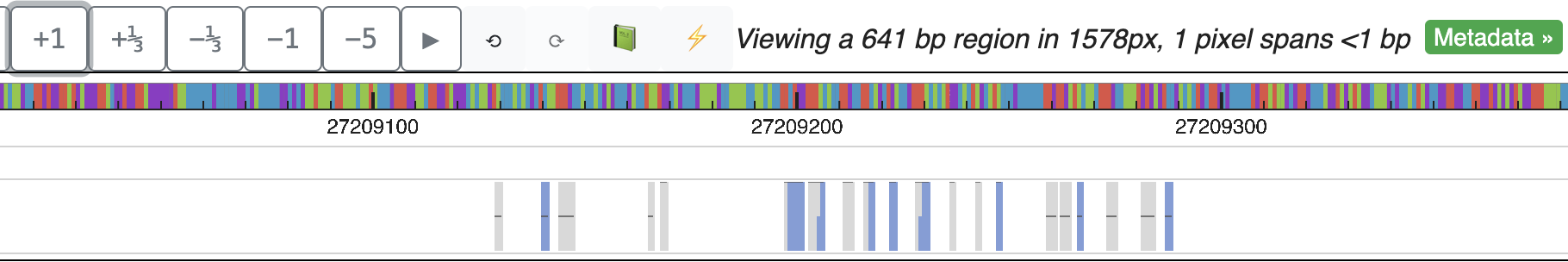 |
| --- |

Each bar represents methylation status of a C base in the genome; a gray bar without blue color means that C is fully unmethylated, whereas a full blue bar means that C base is fully methylated. Black line indicates read depth.
