# Supplementary figures and images for: BAllC and BAllCools: efficient formatting and operating for single-cell DNA methylation data
Source: Bioinformatics. 2024 Jun 21;40(7):btae404. doi: 10.1093/bioinformatics/btae404 (PMC11216754; doi:10.1093/bioinformatics/btae404)

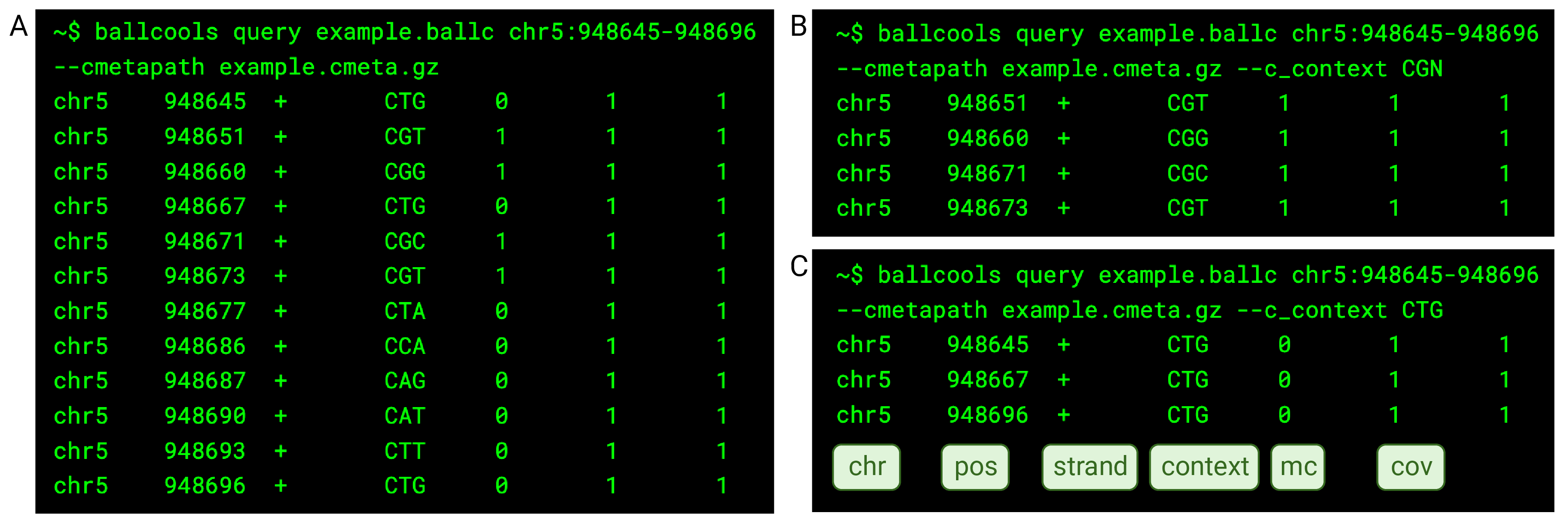

Supplement: btae404_Supplementary_Data [file btae404_supplementary_data.zip › figS2.context-query.png]

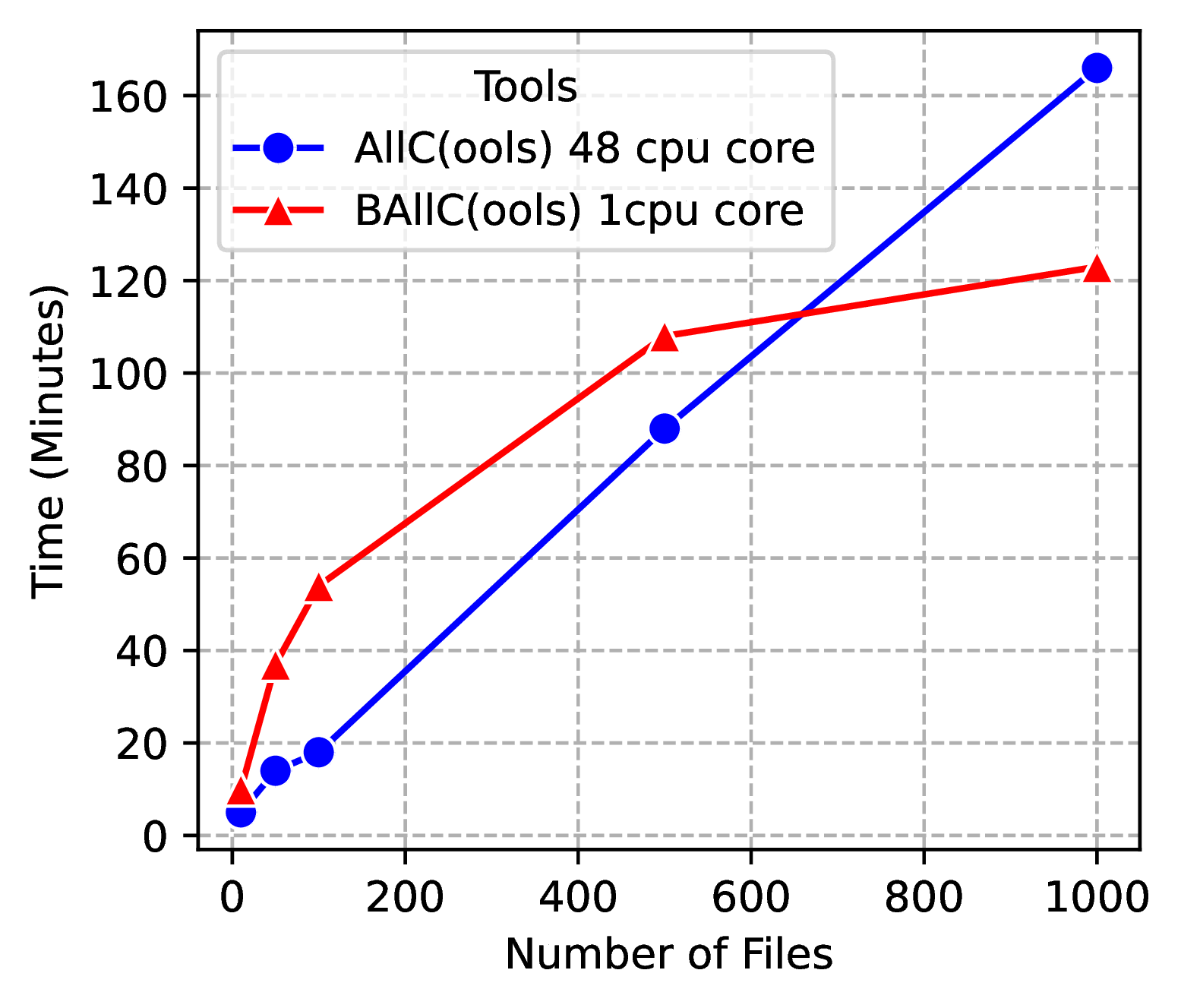

Supplement: btae404_Supplementary_Data [file btae404_supplementary_data.zip › figS1.merging-time.png]

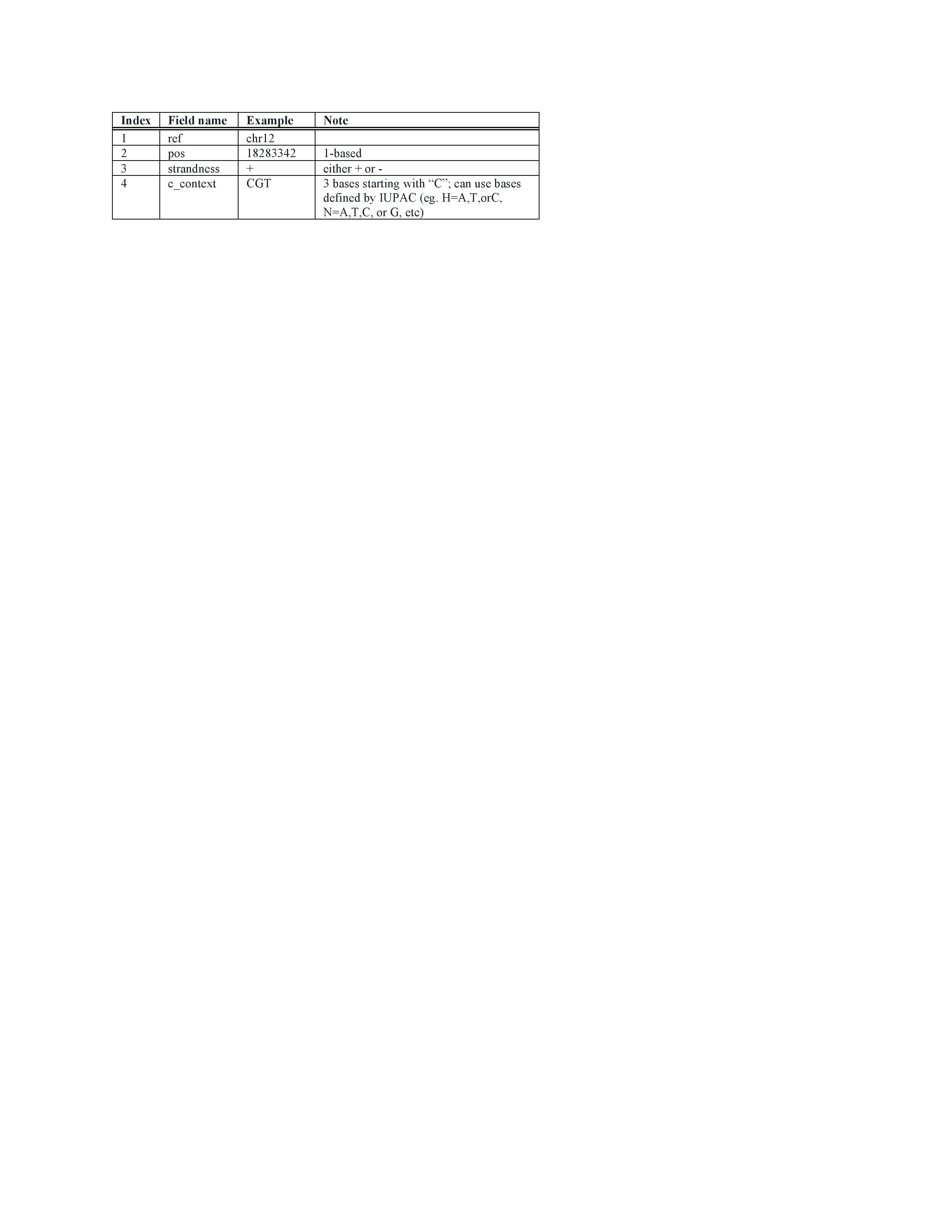

Supplement: btae404_Supplementary_Data [file btae404_supplementary_data.zip › tabS1.ballc-meta-format.png]
